# 

**Published:** 1900-09-29

**Authors:** 


**DEATH.**

Scarth, Margaret Rebecca (Nightingale, and Queen Victoria's Jubilee Nurse), died at the Nursing Institute, Grimsby, September 21st, 1900, age 27. (2,731)

### NOTICE TO CORRESPONDENTS.

All MS., Letters, Books for Review, and other matters intended for the Editor should be addressed The Editor, "The Hospital," The Hospital Building, 28 & 29, Southampton Street, Strand, W.C.

The Editor cannot undertake to return rejected MS., even when accompanied by stamped directed envelope.

**Notice to Advertisers and Subscribers.**

All Advertisements, Orders for Copies of The Hospital, and Business Communications should be addressed to **THE MANAGER (Not to the Editor)**, The Hospital, The Hospital Building, 28 & 29, Southampton Street, Strand, London, W.C.

The Cost of Subscription, post free, is as follows:—Per week, 2½d.; per quarter, 2s. 8d.; per half-year, 5s. 3d.; per annum, 10s. 6d. Foreign:—Per annum, 15s. 2d., payable, in all cases, in advance.

**NOTICE.—Vol. XXVIII. of "The Hospital" (April to September, 1900), in handsome cloth binding, will shortly be on sale at the Office of this Journal, price 7s. 6d. Cases for binding the Numbers contained in this Volume 1s. 6d. Index to Vol. XXVIII., 2½d., post free.**

**The Monthly Part for October will be ready on October 1st. Price 6d., by post 8d.**

### SCRAPS AND GLEANINGS.

DURING 1899 the number of in-patients treated at St. John's Hospital, Limerick, was 642, and the number of out-patients was 1,107.

BOVRIL (Limited) have been awarded two gold medals at the Paris Exhibition; one for their general products, and one for their emergency rations.

THE gross amount of the Hospital Saturday Collection in Belfast this year was £500; in 1899 the amount collected was £650, whilst in 1898 it was £350.

A TENDER amounting to £6,580 odd has been accepted by the Cambridge Town Council for the proposed alterations and additions to the Infectious Diseases Hospital.

THE amount produced by Mr. Studt's recent hospital fête in aid of the Swansea Hospital was £589 odd; the expenses were £371 odd, so that the profits realised amounted to £218.

THE recent fête held on behalf of the Samaritan Hospital for Women and Children, Belfast, produced £325, and in accordance with his generous promise Dr. St. Clair Boyd has cleared off the remainder of the debt by a donation of £125.

THE Little Hoppers' Hospital, which was started three years ago in the Paddock Wood District, is continuing to prove a blessing to the hoppers whose children are attacked with various ailments, and who themselves are treated at the Little Hoppers' Hospital for simple injuries.

DURING the past year the total number of in and out patients treated at the Sheffield Royal Hospital was 23,304. The total ordinary income for the year amounted to £6,750, and legacies were received amounting to £3,464, which latter item was placed to capital account, this account having been increased during the year by £4,024. The expenditure for the year was £7,340.

THERE are two vacancies upon the honorary medical staff of the East Suffolk and Ipswich Hospital, caused by the resignation of two of the senior members of the staff. By a recent resolution of the governors the honorary members of the medical staff are in future to be appointed for a period of 10 years, at the end of which time they must retire, but they will be eligible for re-election.

THERE is a deficit of £247 in the amount required for the purpose of erecting the new laundry machinery at the Swansea Hospital. Of this sum one gentleman has guaranteed to obtain £100 in one year, and it is hoped that the remaining £147 will shortly be provided by the public. This new laundry machinery to be supplied comprises a new heating chamber, provision for working the machinery by electricity, a new working machine, and electric motor.

The first portion of the Royal Alexandra Hospital and Children's Home, at Rhyl, has just been opened. The whole building is being erected at a cost of £40,000 to £50,000, and at least £6,000 is required to complete the next block of buildings.

Saturday, September 29th, is fixed for the Hospital Saturday collection in Cork this year, and it is hoped that the amount then received will compensate in some measure for the lapse of last year's collection. The amount collected in 1898 was £600 9s. 1d.

The proposal of the Boarding-out Committee to erect cottage homes at Torquay in connection with the Newton Abbot Board of Guardians has been referred to a special committee to consider and report upon, in view of another proposal to build the homes on a site adjoining the Newton Abbot Workhouse.

At the annual meeting of the subscribers of the Cottage Home for Convalescents at Newhills, the Rev. Dr. Smith, in the course of his speech, paid special tribute to the help they had received from the members of the legal profession who, he said, had come forward most generously, and who had been most helpful in recommending the home to trustees and also to clients.

At a largely attended meeting held recently at Manchester a resolution emphasising the necessity of establishing a Jewish hospital in Manchester was carried after some opposition on the part of prominent Jews present. A canvassing committee was afterwards appointed. It is desired that a sum of £1,000 should be obtained in the first instance, with which a house capable of accommodating about 30 beds could be secured.

Three hundred and forty fresh cases of out-patients applied for treatment during the month of August at the Kent County Ophthalmic Hospital. At the last monthly meeting of this institution it was decided for the present not to close any of the beds (which it had been feared would be imperative owing to scarcity of funds), but to exercise great care in the admission of in-patients, and to rely upon the public and the supporters of the institution coming forward with increased funds so as to avoid curtailing the beneficial work of the institution.

At a special meeting of the governors and supporters of the Hereford General Infirmary, held for the purpose of considering the financial position, the report of the committee which had been appointed to inquire into this matter was adopted. This report recommends (1) that, in place of the present secretary, a superintendent and secretary should be appointed, who would devote his whole time to the business of the infirmary, and reside in the house; (2) that some small payment should be made by the out-patients; (3) that the subscribers should be urged to exercise more care in the selection of proper cases for admission as patients.

Ex-Provost Moncur has placed at the disposal of the committee a further sum of £5,000 for the Dundee Sanatorium for Consumptives. On the plans being gone into at the commencement of September, it was found that the total estimated cost of the erection and equipment of the building would be between £12,000 and £13,000, which was £3,000 beyond the figure originally named as being required for this purpose, *i.e.*, £10,000, which handsome sum had been at the time handed over most generously to the committee by Ex-Provost Moncur. Thus, Mr. Moncur has given to Dundee a sum of £15,000, which will entirely cover the cost of erection and equipment of the Sanatorium for Consumptives.

### THE STUDENT OF MEDICINE.

### APPOINTMENTS.

R. Belli, M.D., Ch.D., appointed Government Medical Officer and Public Vaccinator at Walgett, New South Wales. S. R. Blake, L.R.C.P., L.R.C.S.Edin., appointed Medical Officer for the No. 4 District of the West Ham Union. G. O. E. Burkitt, L.R.C.P., L.R.C.S.Irl., appointed Honorary Surgeon to the Ear and Throat Department of the Perth Public Hospital, Australia. W. J. Clarke, M.R.C.S.Eng., appointed Medical Officer for the Second District of the Newbury Union. F. W. S. Davies, M.R.C.S.Eng., L.R.C.P.Lond., appointed Honorary Anæsthetist to the Cardiff Infirmary. N. Dodd, L.R.C.P.&S.Edin., appointed Junior Assistant House Surgeon to the Stockport Infirmary. J. H. Finemore, L.R.C.P.Edin., L.S.A., appointed Medical Officer at Boonah, Queensland. B. B. Hoggan, L.R.C.P., L.R.C.S.Edin., L.F.P.S.Glasg., appointed Health Officer for the Shire of Meredith, Victoria. F. M. House, M.R.C.S.Eng., L.R.C.P.Lond., appointed Health Officer to the Beverley Local Board of Health, West Australia. J. Howells, M.R.C.S., L.R.C.P.Lond., appointed Surgeon to the Abercrave Collieries. G. B. D. Macdonald, M.B., Ch.M.Aberd., appointed Health Officer for the Shire of Waranga, Eastern Riding, Victoria. D. A. C. Pearce, M.D.Lond., B.S., M.R.C.S., appointed Medical Officer and Public Vaccinator for the Chartham District of the Bridge Union. E. Pratt, M.D.Lond., appointed Honorary Anæsthetist to the Cardiff Infirmary. R. Rendle, F.R.C.S., appointed Health Officer at Normanton, Queensland. T. S. Robson, M.R.C.S., L.R.C.P.Lond., appointed Medical Officer for the St. Osyth District of the Tendring Union. F. A. Southam, M.B., F.R.C.S., appointed Honorary Surgeon to the Manchester Cancer Pavilion and Home. N. F. Stallard, M.B., M.R.C.S., L.R.C.P., appointed House Surgeon and Registrar to the Royal Orthopædic Hospital. W. H. Thorman, B.A.-Cantab., M.R.C.S., L.R.C.P., appointed Medical Officer and Public Vaccinator for the Kirkburton District of the Huddersfield Union. G. G. Turner, M.B., B.S., M.R.C.S., L.R.C.P., appointed Surgical Registrar to the Royal Infirmary, Newcastle-on-Tyne. Mrs. Mary Hamilton Williams, M.B., B.S.Lond., D.P.H.Cantab., appointed Special Service Medical Officer to the Gambia, 1900, under Colonial Office Special Service Medical Officer on the Staff of the Ashanti Expeditionary Force. C. S. Willis, M.B., appointed Government Medical Officer at Wyalong, New South Wales. C. G. R. Wood, M.R.C.S.Eng., L.R.C.P.-Lond., appointed Surgeon to the Eye, Ear, and Throat Hospital for Shropshire and Wales, Shrewsbury. G. A. Wright, M.B., F.R.C.S., appointed Honorary Surgeon to the Manchester Cancer Pavilion and Home.

28 & 29, SOUTHAMPTON STREET, STRAND, LONDON, W.C.

### THE NURSE'S REPORT BOOK.

Foolscap 4to., Black cover, **6d.** net, post free; or 12 for **4s. 6d.,** post free.

**Compiled by K. H. (Cert.)**

H. J. GLAISHER, 57, Wigmore Street, Cavendish Square, W.

